# Pulmonary Versus Nonpulmonary Mucormycosis: A Comparative Analysis of Clinical Characteristics and Inpatient Outcomes Using the National Inpatient Sample

**DOI:** 10.3390/jof12100789

**Published:** 2026-10-21

**Authors:** Abdirahman Ahmed, Moussa Chehab, Courtney Hagerman, Ayman O. Soubani

**Affiliations:** 1 Detroit Wayne County Health Authority (Authority Health) Graduate Medical Education Consortium, Detroit, MI 48202, USA; abdi.ahmed0823@gmail.com (A.A.); mchehab-gme@authorityhealth.org (M.C.); cdhagerman@gmail.com (C.H.); 2 College of Osteopathic Medicine, Michigan State University, East Lansing, MI 48824, USA; 3 Division of Pulmonary, Critical Care and Sleep Medicine, Wayne State University School of Medicine, Detroit, MI 48202, USA

**Keywords:** mucormycosis, pulmonary mucormycosis, National Inpatient Sample, acute respiratory failure, mechanical ventilation, inpatient mortality

## Abstract

Background: Comparative data on pulmonary and nonpulmonary mucormycosis are limited. Methods: We analyzed adult hospitalizations in the United States Healthcare Cost and Utilization Project National Inpatient Sample from 2018 through 2021. Pulmonary involvement was identified by ICD-10-CM code B46.0. Survey-weighted models adjusted for age, sex, diabetes, hematologic malignancy, and hematopoietic stem cell transplantation; mortality was examined separately with additional illness-severity adjustment. Results: Among 1120 sampled adult hospitalizations, representing approximately 5600 weighted hospitalizations, 308 (27.5%) had pulmonary involvement. Pulmonary cases more often had hematologic malignancy, transplantation, and extreme illness severity while having diabetes less often. Pulmonary involvement was associated with acute respiratory failure defined by J96.0 codes (adjusted odds ratio [aOR], 1.54; 95% confidence interval [CI], 1.14–2.07) and invasive mechanical ventilation (aOR, 1.49; 95% CI, 1.08–2.06). In-hospital mortality did not differ significantly in the crude comparison (24.03% versus 20.57%; *p* = 0.1916), baseline-adjusted model (aOR, 1.11; 95% CI, 0.81–1.53), or model additionally adjusted for illness severity (aOR, 0.96; 95% CI, 0.69–1.34). Respiratory associations persisted after excluding pulmonary cases with concurrent nonpulmonary mucormycosis coding. Conclusions: Pulmonary involvement was associated with greater respiratory morbidity among US hospitalizations with coded mucormycosis. No statistically significant mortality association was detected; these findings do not establish equivalence or a causal effect of infection site.

## 1. Introduction

Mucormycosis is an invasive fungal infection caused by fungi of the order Mucorales and is associated with substantial morbidity and mortality, particularly among immunocompromised patients. Although uncommon, it is among the most aggressive invasive fungal infections because of its angioinvasive nature, rapid tissue destruction, and high mortality despite advances in antifungal therapy and surgical management [1,2]. Established risk factors include hematologic malignancy, hematopoietic stem cell transplantation, solid organ transplantation, prolonged corticosteroid exposure, neutropenia, uncontrolled diabetes mellitus, and other conditions associated with impaired host immunity [2,3].

In US transplant surveillance, *Rhizopus* was the most frequently identified Mucorales genus, followed by *Mucor* [4]. Other recognized causes include *Cunninghamella bertholletiae*, *Lichtheimia*, and *Apophysomyces* species [5]. This transplant-based distribution cannot be assumed to represent every US population, and administrative diagnosis codes do not identify the causative organism.

Clinical manifestations vary according to the site of infection and include rhino–orbital–cerebral, pulmonary, cutaneous, gastrointestinal, and disseminated disease. Pulmonary mucormycosis is the second most common presentation after rhino–orbital–cerebral disease [3] and occurs predominantly among patients with hematologic malignancy, prolonged neutropenia, or hematopoietic stem cell transplantation [3,6,7]. Because pulmonary infection frequently presents with nonspecific respiratory symptoms and can progress rapidly to respiratory failure, delayed diagnosis is common and contributes to poor clinical outcomes [1,2].

Most published evidence consists of case reports, single-center cohorts, and systematic reviews [3,6,7,8]. National comparisons between pulmonary and nonpulmonary disease remain limited. We therefore compared host characteristics, respiratory complications, resource utilization, and inpatient mortality among US adult hospitalizations with coded mucormycosis during 2018–2021.

## 2. Materials and Methods

### 2.1. Study Design and Data Source

We conducted a retrospective cohort study using the 2018–2021 Healthcare Cost and Utilization Project (HCUP) National Inpatient Sample (NIS) [9]. The NIS approximates a 20% stratified sample of discharges from US community hospitals. The unit of analysis was the hospitalization, not an individual patient. Discharge weights were used to estimate the total number of hospitalizations across the four study years, rather than an annual average.

NIS data are de-identified administrative discharge records available through the HCUP Central Distributor under a data use agreement (DUA); access requires HCUP DUA training and signed agreements [10,11]. They are not unrestricted public-use data. No institutional review board approval or exemption determination was obtained for this analysis. The investigators did not access identifiable patient information.

### 2.2. Study Population

Hospitalizations with mucormycosis were identified by ICD-10-CM codes B46.0, B46.1, B46.2, B46.3, B46.4, B46.8, or B46.9 in any of 40 diagnosis fields (I10_DX1–I10_DX40). We restricted the revised analysis to adults aged at least 18 years. The maximum recorded AGE value was 90; HCUP aggregates ages above 89 into the category 90 years or older [10].

Pulmonary involvement was defined by B46.0 in any diagnosis field, including hospitalizations with concurrent nonpulmonary mucormycosis codes. The comparison group included rhinocerebral, gastrointestinal, cutaneous, disseminated, other specified, and unspecified mucormycosis without B46.0. Pulmonary coding took precedence when both groups of codes were present. A sensitivity analysis excluded pulmonary-coded hospitalizations with any concurrent nonpulmonary mucormycosis code while retaining the original nonpulmonary comparison group.

Case identification relied on discharge coding. Culture, histopathology, matrix-assisted laser desorption/ionization time-of-flight mass spectrometry (MALDI-TOF), molecular testing, and imaging findings were not available for case adjudication. We could not classify cases as microbiologically confirmed, probable, or clinically suspected, or determine the positive predictive value of the coding algorithm. Fungal genus and species were unavailable.

### 2.3. Variables and Outcomes

Age, sex, race, primary expected payer, median household income quartile for the patient ZIP code, and hospital location/teaching status were obtained from the NIS. Race was grouped as White, Black, Hispanic, and other; payer as Medicare, Medicaid, private insurance, self-pay, and other. Rural and urban nonteaching hospitals were combined and compared with urban teaching hospitals.

Diagnosis-based comorbidities included diabetes mellitus (E08–E11 and E13), hematologic malignancy (C81–C96), solid malignancy (C00–C80), hematopoietic stem cell transplantation (Z94.84), and solid organ transplantation (Z94.0–Z94.9, excluding Z94.84). These administrative categories do not provide disease activity, treatment intensity, or transplant timing. The supplied All Patient Refined Diagnosis Related Group (APR-DRG) Severity of Illness subclass was collapsed into extreme (4) versus minor, moderate, or major (1–3). APR-DRG Risk of Mortality was grouped as minor/moderate (1–2), major (3), and extreme (4).

The primary outcome was in-hospital mortality. Secondary outcomes were acute respiratory failure, invasive mechanical ventilation, acute kidney injury (N17.x), acute respiratory distress syndrome (J80), and shock (R57.x). The revised primary acute respiratory-failure definition used J96.0x only. A broader sensitivity definition included J96.0x, J96.2x (acute-on-chronic respiratory failure), and J96.9x (unspecified respiratory failure), and is termed expanded respiratory failure. The J96.0-only definition does not capture every acute-on-chronic presentation.

Invasive mechanical ventilation was identified by ICD-10-PCS codes 5A1935Z, 5A1945Z, or 5A1955Z in I10_PR1–I10_PR25. Length of stay (LOS) and total hospital charges (TOTCHG) were taken directly from the NIS. Charges are nominal amounts for each discharge year; we did not apply cost-to-charge ratios or inflation adjustment.

The planned exploratory analysis of discharge disposition was restricted to survivors. Categories were routine discharge (DISPUNIFORM = 1), transfer/other/against medical advice (2, 5, or 7), and home healthcare (6). Unknown disposition (99) was excluded from the survey comparison. The combined transfer/other category must not be interpreted as facility transfers alone.

### 2.4. Statistical Analysis

All survey analyses incorporated DISCWT, hospital clustering, and NIS stratification. For pooled years, we defined distinct hospital-by-year primary sampling units and stratum-by-year strata from HOSP_NIS, NIS_STRATUM, and YEAR. Variances were estimated by Taylor linearization with a single unit (centered). Adult mucormycosis and survivor analyses used survey subpopulation estimation, retaining records outside the domain for variance estimation.

Tables report unweighted sample counts and survey-weighted percentages. Continuous variables are presented as survey-weighted means with unweighted standard deviations; selected medians and interquartile ranges are descriptive and unweighted. Categorical comparisons used survey-adjusted Pearson tests with the design-based F approximation (Rao–Scott adjustment); continuous comparisons used survey-weighted linear regression. Percentages for variables with missing values use non-missing denominators. We did not impute missing data.

Survey-weighted logistic models adjusted for age, sex, diabetes, hematologic malignancy, and hematopoietic stem cell transplantation, retaining the clinically selected baseline covariates from the original analysis. Baseline-adjusted mortality was the principal mortality model; a separate model added extreme APR-DRG severity as a sensitivity analysis because severity incorporates information accrued during hospitalization and may overlap with outcomes. Severity was not included in respiratory outcome models. The same models were fitted after exclusion of pulmonary-coded hospitalizations with concurrent nonpulmonary codes; expanded respiratory failure was examined in both cohorts.

Adjusted odds ratios (aORs) and 95% confidence intervals (CIs) are reported. Tests were two-sided with nominal *p* < 0.05; no multiplicity correction was applied. Secondary and exploratory comparisons, particularly those near this threshold, require cautious interpretation. Analyses were performed in Stata 19.5. The revision corrected the adult cohort restriction, used year-specific survey identifiers, and replaced the primary respiratory-failure definition with J96.0-only coding; all reported analyses were refreshed accordingly.

## 3. Results

### 3.1. Cohort and Clinical Characteristics

Of 1221 sampled mucormycosis hospitalizations, 101 were younger than 18 years and were excluded. The revised cohort comprised 1120 adult hospitalizations: 308 (27.5%) with pulmonary involvement and 812 (72.5%) without pulmonary coding, representing approximately 5600 weighted hospitalizations during 2018–2021. Adult sample counts by year were 242, 259, 264, and 355, respectively.

Mean age did not differ significantly between pulmonary and nonpulmonary groups (55.23 versus 54.06 years; *p* = 0.2276). Male sex was recorded in 64.61% versus 60.47% (*p* = 0.2179 for sex distribution). Pulmonary cases had more hematologic malignancy (39.29% versus 21.80%; *p* < 0.001), hematopoietic stem cell transplantation (6.17% versus 3.08%; *p* = 0.0274), and solid organ transplantation (10.06% versus 6.16%; *p* = 0.0345), and less diabetes (39.94% versus 54.19%; *p* < 0.001). Extreme illness severity was more frequent with pulmonary coding (77.60% versus 64.29%; *p* < 0.001). The overall risk-of-mortality distribution differed (*p* = 0.0056). Other demographic comparisons are shown in Table 1.

### 3.2. Inpatient Outcomes

Acute respiratory failure defined by J96.0 occurred in 34.09% of pulmonary and 24.88% of nonpulmonary hospitalizations (*p* = 0.0024). Mechanical ventilation occurred in 27.92% versus 22.04% (*p* = 0.0453). In-hospital mortality did not differ significantly (24.03% versus 20.57%; *p* = 0.1916). Shock was recorded in 6.82% versus 4.06% (*p* = 0.0473), whereas acute kidney injury and acute respiratory distress syndrome did not differ significantly (Table 2).

Mean LOS was 21.22 versus 21.02 days (*p* = 0.8970). Mean nominal hospital charges were $441,181 versus $490,069 (*p* = 0.2256). The survivor disposition distribution differed overall (*p* = 0.0218); routine discharge, transfer/other/against medical advice, and home healthcare accounted for 44.87%, 25.21%, and 29.91% of pulmonary survivors with known disposition, compared with 39.75%, 35.09%, and 25.16% of nonpulmonary survivors. This is an exploratory distributional comparison, not evidence of different rehabilitation requirements or functional recovery.

### 3.3. Adjusted Models and Sensitivity Analyses

After baseline adjustment, pulmonary involvement was associated with acute respiratory failure (aOR, 1.54; 95% CI, 1.14–2.07; *p* = 0.005) and mechanical ventilation (aOR, 1.49; 95% CI, 1.08–2.06; *p* = 0.016). Complete covariate results are provided in Table 3.

No statistically significant pulmonary mortality association was detected in the baseline-adjusted model (aOR, 1.11; 95% CI, 0.81–1.53; *p* = 0.523) or the separate model adding extreme illness severity (aOR, 0.96; 95% CI, 0.69–1.34; *p* = 0.818). Extreme severity was strongly associated with death (aOR, 13.96; 95% CI, 7.47–26.09; *p* < 0.001). Hematologic malignancy and stem cell transplantation were not significantly associated with inpatient death in either mortality model; their confidence intervals and other covariate results are shown in Table 4.

**Table 3:** Respiratory outcome models in the primary adult cohort.

Covariate	ARF aOR (95% CI)	*p*	IMV aOR (95% CI)	*p*
Pulmonary involvement	1.54 (1.14–2.07)	0.005	1.49 (1.08–2.06)	0.016
Age, per year	0.99 (0.98–1.00)	0.107	0.99 (0.98–1.00)	0.009
Female sex	0.89 (0.68–1.17)	0.411	0.72 (0.54–0.97)	0.030
Diabetes	0.73 (0.55–0.98)	0.035	0.71 (0.53–0.96)	0.027
Hematologic malignancy	0.93 (0.69–1.26)	0.642	0.49 (0.34–0.72)	<0.001
Stem cell transplant	0.77 (0.38–1.55)	0.461	0.88 (0.40–1.92)	0.750

Both models include all 1120 adult hospitalizations, adjusting for the listed baseline covariates without APR-DRG severity. ARF uses J96.0 only. Reference categories are as in Table 4. ARF, acute respiratory failure; IMV, invasive mechanical ventilation.

Excluding 17 pulmonary-coded hospitalizations with concurrent nonpulmonary mucormycosis codes left 291 isolated pulmonary and 812 nonpulmonary hospitalizations (total *n* = 1103). Associations persisted for acute respiratory failure (aOR, 1.58; 95% CI, 1.17–2.14; *p* = 0.003) and ventilation (aOR, 1.45; 95% CI, 1.05–2.02; *p* = 0.026). Mortality remained nonsignificant in the baseline model (aOR, 1.04; 95% CI, 0.75–1.44; *p* = 0.808) and severity-adjusted model (aOR, 0.91; 95% CI, 0.64–1.27; *p* = 0.570).

The expanded respiratory-failure definition identified 383 adult hospitalizations, including 307 with J96.0 codes and, among those without J96.0, 57 with J96.2 and 19 with J96.9. Pulmonary involvement was associated with this expanded outcome in the primary cohort (aOR, 1.64; 95% CI, 1.23–2.19; *p* = 0.001) and isolated-pulmonary sensitivity cohort (aOR, 1.65; 95% CI, 1.23–2.22; *p* = 0.001). Table 5 summarizes all sensitivity estimates.

## 4. Discussion

Pulmonary involvement was associated with greater respiratory morbidity in this national sample of US adult hospitalizations with coded mucormycosis. Associations with J96.0-defined acute respiratory failure and mechanical ventilation persisted after excluding concurrent nonpulmonary mucormycosis coding. No statistically significant mortality association was detected in the crude, baseline-adjusted, or severity-adjusted analyses. These results do not demonstrate equivalent mortality or establish that infection site has no causal effect.

The pulmonary group had a malignancy- and transplant-associated host profile, whereas diabetes was more common in nonpulmonary disease. This is consistent with prior descriptions of variation in anatomic presentation by host condition [3,6,7]. Unlike the original all-age results, age was not significantly different in the corrected adult cohort. The solid organ transplantation difference also warrants acknowledgment, although it was not included in the retained baseline model and may contribute to residual confounding.

Host characteristics describe only part of the host–pathogen interaction. Mucorales angioinvasion can produce thrombosis and tissue necrosis, and organism biology and antifungal susceptibility may influence disease progression and treatment response [1,2]. US transplant surveillance identified *Rhizopus* and *Mucor* as prominent genera [4], but species distributions in this NIS cohort cannot be inferred from those external data. Without genus, species, or susceptibility results, we could not examine pathogen-specific associations or separate their contribution from host factors.

Detailed clinical cohorts and pooled case series provide diagnostic, treatment, and prognostic information that is unavailable in discharge data [7,8,12]. For example, Powell and colleagues reported associations between surgical selection, survival to discharge, and underlying comorbidity in a single-center pulmonary cohort [12]. Those findings address different populations and endpoints; we therefore avoid direct numerical mortality comparisons with our administrative cohort. Referral patterns, diagnostic confirmation, treatment selection, and follow-up can all affect comparability.

The nonsignificant mortality estimates for hematologic malignancy and stem cell transplantation should not be interpreted as absence of clinical risk. Transplant cases were few, with wide confidence intervals. Codes do not distinguish malignancy activity, treatment exposure, neutropenia severity or duration, transplant type, or time since transplantation. Selection into an already affected hospitalized cohort and residual confounding may further alter associations. The baseline model also did not detect these associations, so the findings cannot be attributed solely to APR-DRG adjustment; inpatient mortality is distinct from long-term survival assessed in some clinical cohorts.

Diabetes was associated with lower adjusted odds of death and respiratory outcomes, but these observational findings do not show a protective effect. Differences in host phenotype, infection site, diabetes severity, competing comorbidities, and unmeasured treatment factors may explain the pattern. APR-DRG severity reflects information accumulated during hospitalization rather than severity solely at admission. Its strong mortality association is therefore descriptive, and additional adjustment may introduce overadjustment or other bias. The separate baseline mortality model addresses this concern without establishing a causal infection-site effect.

Discharge disposition was a planned exploratory analysis restricted to survivors, not a post hoc analysis. The overall distribution differed, but conditioning on survival and combining transfers with other/against-medical-advice discharges limit interpretation. These findings generate hypotheses rather than demonstrate differences in post-discharge respiratory care, rehabilitation, or function. Shock and crude ventilation comparisons were close to the nominal significance threshold and should also be interpreted cautiously given multiple secondary comparisons.

Several limitations constrain inference. Mucormycosis was identified by discharge coding without microbiologic, histopathologic, or imaging adjudication; the coding algorithm’s positive predictive value in this dataset is unknown. Confirmed infection cannot be separated from suspected or miscoded disease. The nonpulmonary group is heterogeneous, encompassing rhinocerebral, gastrointestinal, cutaneous, disseminated, other, and unspecified forms with different prognoses. The isolated-pulmonary sensitivity analysis addresses concurrent coding but does not remove heterogeneity in the comparison group.

Baseline adjustment was limited to selected coded covariates. Prolonged corticosteroid exposure, cumulative dose, neutropenia severity and duration, malignancy activity, transplant details, laboratory abnormalities, and diagnostic delay were not assessed. The analysis also lacked antifungal agent, dose, duration, susceptibility, combination therapy, and treatment-initiation timing. Although the NIS contains procedure codes, disease-directed surgical resection, indication, and treatment timing were not ascertained in this study. We therefore cannot evaluate treatment effectiveness, distinguish effects of delayed diagnosis, or fully control treatment-related confounding.

The NIS captures hospitalizations and does not permit longitudinal identification of individual patients in this analysis. Repeat admissions, post-discharge death, relapse, and long-term survival could not be assessed. Findings apply to US adult inpatient discharges and may not generalize to children, outpatient disease, non-US settings, or populations with different pathogen distributions and treatment access. Charges were not converted to costs or adjusted for inflation, and they were highly variable. Missing race, income, charges, and unknown disposition were handled with non-missing denominators. Residual confounding and coding error remain possible despite the sensitivity analyses.

## 5. Conclusions

Pulmonary involvement was associated with acute respiratory failure and mechanical ventilation among US adult hospitalizations with coded mucormycosis, including after exclusion of concurrent nonpulmonary coding. No statistically significant association with in-hospital mortality was detected in either the baseline-adjusted model or the separate severity-adjusted model. The confidence intervals, unmeasured host and treatment factors, and limitations of discharge coding preclude conclusions of equivalent mortality or causal independence of infection site.

## Figures and Tables

**Table 1:** Characteristics of adult mucormycosis hospitalizations.

Characteristic	Nonpulmonary *n* = 812	Pulmonary *n* = 308	Survey *p*
Age, mean (SD), years	54.06 (15.57)	55.23 (14.52)	0.2276
Male	491 (60.47)	199 (64.61)	0.2179
Female	321 (39.53)	109 (35.39)	
Race			0.2792
White	421 (53.91)	159 (53.72)	
Black	111 (14.21)	55 (18.58)	
Hispanic	176 (22.54)	61 (20.61)	
Other race	73 (9.35)	21 (7.10)	
Primary payer			0.2219
Medicare	268 (33.00)	119 (38.64)	
Medicaid	212 (26.11)	70 (22.73)	
Private insurance	245 (30.17)	95 (30.84)	
Self-pay	51 (6.28)	11 (3.57)	
Other payer	36 (4.43)	13 (4.22)	
ZIP-code income quartile			0.1216
Quartile 1	236 (30.03)	80 (26.14)	
Quartile 2	206 (26.21)	66 (21.57)	
Quartile 3	193 (24.55)	89 (29.08)	
Quartile 4	151 (19.21)	71 (23.20)	
Hospital teaching status			0.1806
Rural or urban nonteaching	115 (14.16)	34 (11.04)	
Urban teaching	697 (85.84)	274 (88.96)	
Diabetes	440 (54.19)	123 (39.94)	<0.001
Hematologic malignancy	177 (21.80)	121 (39.29)	<0.001
Solid malignancy	39 (4.80)	18 (5.84)	0.5097
Hematopoietic stem cell transplant	25 (3.08)	19 (6.17)	0.0274
Solid organ transplant	50 (6.16)	31 (10.06)	0.0345
Extreme APR-DRG illness severity	522 (64.29)	239 (77.60)	<0.001
APR-DRG risk of mortality			0.0056
Minor or moderate	88 (10.84)	14 (4.55)	
Major	329 (40.52)	133 (43.18)	
Extreme	395 (48.65)	161 (52.27)	

Counts are unweighted; percentages are survey-weighted. Age means are weighted and SDs unweighted. Race was missing in 43 and income in 28 hospitalizations; percentages use non-missing denominators. *p* values are overall tests for multicategory variables. APR-DRG, All Patient Refined Diagnosis Related Group.

**Table 2:** Inpatient outcomes and survivor disposition.

Outcome	Nonpulmonary *n* = 812	Pulmonary *n* = 308	Survey *p*
Acute respiratory failure, J96.0	202 (24.88)	105 (34.09)	0.0024
Invasive mechanical ventilation	179 (22.04)	86 (27.92)	0.0453
In-hospital mortality	167 (20.57)	74 (24.03)	0.1916
Acute kidney injury	422 (51.97)	147 (47.73)	0.2214
Shock	33 (4.06)	21 (6.82)	0.0473
Acute respiratory distress syndrome	26 (3.20)	14 (4.55)	0.2803
LOS, mean (SD), days	21.02 (23.76)	21.22 (22.84)	0.8970
LOS, median [IQR], days	13 [5.5–29]	13 [6–29]	—
Hospital charges, mean (SD), $	490,069 (788,981)	441,181 (555,579)	0.2256
Hospital charges, median [IQR], $	208,929 [71,163,169–533]	222,669 [80,536–583,602]	—
Survivor disposition			0.0218
Routine	256 (39.75)	105 (44.87)	
Transfer or other/AMA	226 (35.09)	59 (25.21)	
Home healthcare	162 (25.16)	70 (29.91)	

Counts are unweighted and percentages survey-weighted. Continuous means are weighted, SDs and medians/IQRs unweighted. Charges are nominal hospital charges, not costs; non-missing charges *n* = 802 and *n* = 303. Disposition percentages exclude unknown values and use known survivors *n* = 644 and *n* = 234. Disposition *p* is an overall test. AMA, against medical advice; IQR, interquartile range; LOS, length of stay. Median rows have no inferential comparison.

**Table 4:** Mortality models in the primary adult cohort.

Covariate	Baseline aOR (95% CI)	*p*	Plus Severity aOR (95% CI)	*p*
Pulmonary involvement	1.11 (0.81–1.53)	0.523	0.96 (0.69–1.34)	0.818
Age, per year	1.01 (1.00–1.02)	0.054	1.01 (1.00–1.02)	0.014
Female sex	0.83 (0.62–1.11)	0.207	0.83 (0.61–1.12)	0.216
Diabetes	0.54 (0.40–0.74)	<0.001	0.60 (0.43–0.84)	0.002
Hematologic malignancy	1.01 (0.72–1.42)	0.942	0.80 (0.56–1.14)	0.218
Stem cell transplant	0.78 (0.38–1.62)	0.505	0.65 (0.31–1.34)	0.242
Extreme illness severity	—	—	13.96 (7.47–26.09)	<0.001

Both models include all 1120 adult hospitalizations and use the year-specific survey design. Reference categories are nonpulmonary, male, and absence of each binary condition. Extreme severity compares subclass 4 with 1–3. aOR, adjusted odds ratio; CI, confidence interval.

**Table 5:** Pulmonary involvement associations across sensitivity analyses.

Outcome/Model	Primary aOR (95% CI)	*p*	Isolated Pulmonary aOR (95% CI)	*p*
Strict ARF	1.54 (1.14–2.07)	0.005	1.58 (1.17–2.14)	0.003
Expanded respiratory failure	1.64 (1.23–2.19)	0.001	1.65 (1.23–2.22)	0.001
Mechanical ventilation	1.49 (1.08–2.06)	0.016	1.45 (1.05–2.02)	0.026
Mortality, baseline adjustment	1.11 (0.81–1.53)	0.523	1.04 (0.75–1.44)	0.808
Mortality, plus severity	0.96 (0.69–1.34)	0.818	0.91 (0.64–1.27)	0.570

Primary cohort *n* = 1120 (308 pulmonary, 812 nonpulmonary). Sensitivity cohort *n* = 1103 (291 isolated pulmonary, 812 nonpulmonary), excluding 17 pulmonary cases with concurrent nonpulmonary mucormycosis coding. Expanded respiratory failure comprises J96.0, J96.2, and J96.9. All models retain baseline covariates; severity is added only in the indicated mortality model.

## Data Availability

The NIS is available through the HCUP Central Distributor subject to licensing, DUA training, and signed data use agreements. The underlying discharge-level data cannot be redistributed by the authors. Access information is available at https://hcup-us.ahrq.gov/nisoverview.jsp.
